# Greater Emotional Gain from Giving in Older Adults: Age-Related Positivity Bias in Charitable Giving

**DOI:** 10.3389/fpsyg.2016.00846

**Published:** 2016-06-15

**Authors:** Pär Bjälkebring, Daniel Västfjäll, Stephan Dickert, Paul Slovic

**Affiliations:** ^1^Department of Psychology, University of GothenburgGothenburg, Sweden; ^2^Linköping UniversityLinköping, Sweden; ^3^Decision Research, University of Oregon, EugeneOR, USA; ^4^Vienna University of Economics and BusinessVienna, Austria

**Keywords:** charitable giving age, emotion, motivation, decision making

## Abstract

Older adults have been shown to avoid negative and prefer positive information to a higher extent than younger adults. This positivity bias influences their information processing as well as decision-making. We investigate age-related positivity bias in charitable giving in two studies. In Study 1 we examine motivational factors in monetary donations, while Study 2 focuses on the emotional effect of actual monetary donations. In Study 1, participants (*n* = 353, age range 20–74 years) were asked to rate their affect toward a person in need and then state how much money they would be willing to donate to help this person. In Study 2, participants (*n* = 108, age range 19–89) were asked to rate their affect toward a donation made a few days prior. Regression analysis was used to investigate whether or not the positivity bias influences the relationship between affect and donations. In Study 1, we found that older adults felt more sympathy and compassion and were less motivated by negative affect when compared to younger adults, who were motivated by both negative and positive affect. In Study 2, we found that the level of positive emotional reactions from monetary donations was higher in older participants compared to younger participants. We find support for an age-related positivity bias in charitable giving. This is true for motivation to make a future donation, as well as affective thinking about a previous donation. We conclude that older adults draw more positive affect from both the planning and outcome of monetary donations and hence benefit more from engaging in monetary charity than their younger counterparts.

## Introduction

Prosocial behavior has been shown to have positive hedonic benefits for both the recipient and the giver. For instance, recent research has shown that people who donate money experience more happiness (Dunn et al., 2008) and, in brain imaging studies, voluntary giving has been associated with activation of the reward centers in the brain (ventral striatum; Harbaugh et al., 2007). The good feeling experienced after doing something good for someone else has been linked to an emotional state of “warm glow” (Andreoni, 1990). Given all the benefits of prosocial behavior, one would expect that behaving prosocially is the rule rather than the exception in people’s daily lives (Dickert et al., 2012). Indeed, a worldwide survey of giving showed that 29% of the respondents donated money, 21% volunteered time, and 47% helped strangers (World Giving Index, 2011). Experimental and field studies provide ample evidence that people do behave prosocially in a variety of contexts and for many different reasons (e.g., Landry et al., 2010; Bekkers and Wiepking, 2011; Slovic et al., 2012), but we know surprisingly little about age differences in the emotional response to prosocial behavior or age differences in emotional motivations for prosocial behavior. For example, in a recent and comprehensive survey of more than 500 articles on charitable giving, age as a factor for giving is almost completely ignored (Bekkers and Wiepking, 2011). Moreover, empirical studies on the underlying motivation for donations highlight affective and cognitive psychological processes (e.g., Kogut and Ritov, 2005; Small et al., 2007; Dickert et al., 2011b; Cryder et al., 2013), but pay very little attention to how these motivations might shift with age.

Prosocial behavior has been shown to increase in middle-age and thereafter, and prosocial acts have been shown to be correlated to well-being in younger, middle-aged and older adults (McAdams et al., 1993). Midlarsky and Hannah (1989) showed, in a sample of people between 5 and 75+ years of age, that older participants were more likely to donate money, when controlling for income, compared to younger adults. They also suggest that prosocial behavior helps enhance the sense of meaning and value in one’s life, as well as increasing perceived competence. They hypothesize that this is especially important for older adults and may have greater impact on well-being in older than compared to younger adults (Midlarsky and Kahana, 1994). Generosity has also been shown to increase with age, and it has been suggested that it is a core aspect of well-being in later life (Kahana et al., 1987; Keyes and Ryff, 1998). In the Bonn Longitudinal Study of Aging, Rudinger and Thomae (1993) showed a connection between prosocial behavior in the family and satisfaction with family life, together with well-being. They also concluded that these prosocial behaviors refer to certain patterns of behavior, rather than global personality disputations or traits. Thus, engaging in proscosial behaviors seems to be part of what has been called “successful aging.”

A possible explanation of these findings may lie in how older adults derive affective feelings from behaviors. Research suggest that older adults engage in behaviors that promotes positive emotional experiences (Carstensen, 1995). This positivity bias may arise in two different ways. First, it may result from positivity enhancements (i.e., greater facilitation in cognitive processing of positive over negative or neutral information in older, compared to younger adults) (Carstensen, 1992; Carstensen et al., 2000, 2011). Second, it may result from negativity reductions, such as decreased cognitive processing of negative compared to positive or neutral material, in older relative to younger adults (Tomaszczyk and Fernandes, 2013). Positivity bias (both positivity enhancements and negativity reductions) have been demonstrated using a variety of tasks in different areas of psychology. Here, older adults have been shown to avoid negative memories and prefer positive memories (Kennedy et al., 2004; Mather and Carstensen, 2005; Tomaszczyk et al., 2008). Older adults also pay more attention to positive information and pay less attention to negative information (Isaacowitz et al., 2006; Orgeta, 2011). In addition, when making decisions, older adults avoid alternatives that are associated with negative affect and prefer those associated with positive affect (Mather and Johnson, 2000; Mather et al., 2004; Löckenhoff and Carstensen, 2007; Kim et al., 2008). These findings suggest that positive and negative affect (and their motivational aspects) may play different roles in prosocial decision making across one’s lifespan.

The present research examines if older adults (compared to younger adults) derive more positive affect from acting prosocially, and if the positive affective consequences of acting prosocially is the main determinant of behavior. Study 1 investigates the underlying affective motivations for charitable giving and Study 2 examines the emotional consequences of having acted prosocially. This enables a closer look at the affective determinants for charitable giving as well as the effect of giving on people’s affective experiences. In line with motivational lifespan theories, we expected that older adults would experience more positive affect from giving, as they value goals related to giving to a higher extent than younger adults (Kennedy et al., 2004; Mather and Carstensen, 2005; Tomaszczyk et al., 2008). Further, in line with the positivity bias, we expected that older adults would be motivated more by positive information than negative information when compared to younger adults (Isaacowitz et al., 2006; Orgeta, 2011).

## Study 1

### Participants

Three-hundred and fifty-three participants of the Decision Research participants pool were recruited by email to take part in an online experiment (age range 20–74 years, *M*_age_ = 47 years, 49.9% female). Of participants 17% were in their 20s, 22% were in their thirties, 28% were in their forties, 20% were in their 50s, 11% were in their 60s and 1% were in their 70s.

### Design and Procedure

Participants were asked to complete a series of unrelated tasks before they were presented with the donation task of the current study, first participants answered questions about age, education, gender, background affect, as well as information regarding giving money to help various organizations. Participants were then informed that we were interested in charitable giving and that they would now see a scenario about a child in need of aid. They were asked to think about feelings such as “warm glow,” a positive feeling that you may experience when you do something good for someone. They were then shown a picture of a single child and her name. Participants were instructed that “This child is in need of aid. The child is facing starvation and is in immediate need of food.” Suppose you are now given the opportunity to donate money to a trusted aid organization to help this child. They were then asked to rate in what extent they felt six different feelings toward this child and also to rate what amount (if any) they wanted to donate.

### Measures

To measure participants’ affective reactions to the child in need we measured how much “Warm Glow” they felt on a 0 (no warm glow) to100 (very strong warm glow) scale. In addition we measured sympathy, compassion, worry, upset, and sadness on a 1–7 point scale. While “warm glow,” worry, upset, and sad can be considered as more purely affective processes, sympathy and compassion are a mix of affective and cognitive processes (Eisenberg and Strayer, 1987). Additionally, we wanted to avoid asking participants if they felt happy when viewing a picture of a child sick child. However, sympathy and compassion are more normative reactions to seeing someone in need, and will be considered affective components of the decision process in this study. In addition to these positive and negative affective components, we asked participants how much they would be willing to donate ($) to help the person in need on a 0–50 scale. Donation amounts were hypothetical in Study 1.

### Results

We wanted to investigate emotional influences on motivational behavior for charitable giving. As seen from the correlational analyses between age and the emotional items (**Table 1**), older adults felt more sympathy and compassion for the child, compared to younger adults. However, there were no age related differences in “Warm Glow,” Worried, Upset, Sad or the amount donated. The donations participants said they would be willing to donate differenced substantially between individuals from $0 to $50, the mean amount participants said they would be willing to donate was $20.68 with a standard deviation of $16.3. In addition, all affective items (both positive and negative) are associated with increase giving. Though, older adults feel more sympathy and compassion than younger adults, there is no difference in the amount donated between younger and older adults. This difference in emotion but not in giving suggests that older adults are less motivated by either positive or negative emotions when compared to younger adults.

**Table 1:** Correlations between variables in study, ^∗^*p* < 0.05, ^∗∗^*p* < 0.01 (*n* = 353).

	Age	Warm Glow	Sympathy	Compassion	Worried	Upset	Sad
Age	1						
WarmGlow	-0.076	1					
Sympathy	0.155ˆ**	0.530^∗∗^	1				
Compassion	0.142ˆ**	0.551^∗∗^	0.937^∗∗^	1			
Worried	0.033	0.516^∗∗^	0.682^∗∗^	0.686^∗∗^	1		
Upset	0.034	0.514^∗∗^	0.703^∗∗^	0.704^∗∗^	0.897^∗∗^	1	
Sad	0.072	0.552^∗∗^	0.817^∗∗^	0.797^∗∗^	0.809^∗∗^	0.846^∗∗^	1
$ Donation amount	-0.027	0.553^∗∗^	0.440^∗∗^	0.481^∗∗^	0.502^∗∗^	0.500^∗∗^	0.475^∗∗^


To investigate this, we *z*-scored and created a mean index from the three positive emotional items (“Warm Glow,” Sympathy and Compassion, α = 0.86) as well as *z*-scored and created a mean index for the three negative emotional items (Worried, Upset and Sad, α = 0.95). When assessed in a factor analysis, the negative and positive items separated into three factors (for analysis see Supplementary Material). The three negative items factored well together, however, compassion and sympathy factored well together and warm glow factored alone. This indicates that warm glow is separated from sympathy and compassion, however, qualitatively warm glow is closer to sympathy and compassion, and using warm glow as a part of the positive affect index or as a separate factor does not change the outcome of the analysis.

**FIGURE 1:**
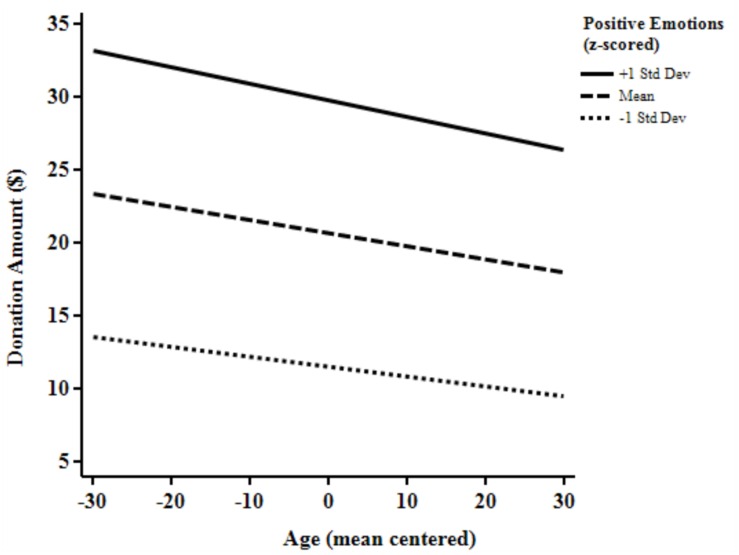
**Visualization of the influence of age and positive emotions on donation amounts (*n* = 353)**.

**FIGURE 2:**
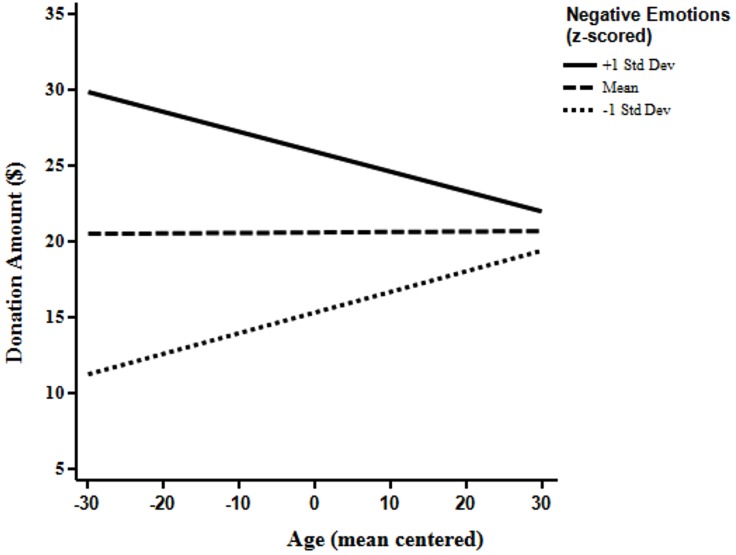
**Visualization of the influence of age and negative emotions on donation amounts (*n* = 353)**.

Using the *z*-scored affective indexes as well as age mean centered at 47 years as predictors of donation amount in dollars. In two separate regression analyses, we investigated whether the motivational influence on donations of positive emotions, as well as negative emotions, differed between younger and older adults. As seen in from the non-significant interaction (*b* = -0.026, *p* < 0.73) between age and positive emotion in the left part of **Table 2** and **Figure 1**, positive emotions did not predict donations in younger and older adults differently, [*R*^2^ = 0.31, *F*(3,348) = 53.0, *p* < 0.01]. However, the interaction between age and negative emotions (*b* = -0.13, *p* = 0.04) shows that negative emotions had a different influence on older and younger adults’ donations [*R*^2^ = 0.28, *F*(3,348) = 45.6, *p* < 0.01; see **Figure 2**]. Negative emotions were a better predictor for younger adults as higher levels of negative emotions corresponded to higher amounts of giving and lower levels of negative emotions correspond to lower amounts of giving. While, for older adults, negative emotions were a much weaker predictor of donation amounts.

**Table 2:** Regression model with continuous age (mean centered) and continuous negative as well as positive affect (*z*-scored) and their interaction predicting donation amount in $ (*n* = 353).

	Positive emotions				Negative emotions			
		
	β	*b*	*SE*	*p*	β	*b*	*SE*	*p*
Age	-0.07	-0.091	0.059	0.12	-0.04	0.059	0.059	0.32
Emotion	0.056	10.322	0.821	<0.001	0.053	9.148	0.784	<0.001
Interaction	-0.02	-0.026	0.076	0.73	-0.10	-0.134	0.066	0.042
R2		0.31	–	<0.001		0.28	–	<0.001


### Discussion

In prior research monetary donations to charitable causes have been associated with both negative emotions (evoked by seeing someone in need), and positive emotions (being able to help this person in need; e.g., Batson, 1990; Kogut and Ritov, 2005; Loewenstein and Small, 2007; Small et al., 2007; Dickert et al., 2011b; Rubaltelli and Agnoli, 2012). In line with these findings, our results show that both the positive and negative affects had positive associations with the amount donated. These findings are also congruent with the notion that mixed emotions may be important in motivating prosocial behavior (Västfjäll et al., unpublished manuscript). However, when comparing older and younger adults in their respective affective response to the person in need, results showed that age was positively correlated with sympathy and compassion, suggesting that as people get older they derive more positive affect from donation scenarios. This finding is in line with previous research on age-related positivity bias that has shown older adults draw more positive affect from material compared to younger adults (Kennedy et al., 2004; Mather and Carstensen, 2005; Tomaszczyk et al., 2008). However, from the correlational analyses no age related differences were shown in how much worry, sadness or, how upset was from seeing the child in need. Therefore, our results support the notion of a positivity enhancement and that the older adults seem to maximize positive affect, rather than minimizing negative emotions with regard to the stimuli.

In addition, younger and older donated similar amounts. As the positive affect for a person was associated with higher donations, we expected that older adults would donate more money, because they felt more positive affect. However, the higher level of positive affect reported by older adults did not motivate them to donate more money than younger adults. This suggests that there are differences in how affect is associated with motivation between older and younger adults. However, our analysis showed that the difference in motivation sprung from the negative emotions rather from the positive emotions. The significant interaction between negative affect and age showed that negative emotions are a stronger motivator for monetary donations in younger adults when compared to older adults. This is line with the negativity reductions in the positivity bias, and relates to research by Tomaszczyk and Fernandes (2013), in which they showed decreased cognitive processing of negative compared to positive material, in older relative to younger adults. The older adults in our sample felt sad, upset and worried when seeing the child in need, however, their donation amount were less influenced by these emotions, this indicates that they avoid processing these emotions.

## Study 2

In Study 1 the affective determinants of prosocial choices were examined. In Study 2 we instead examine the affective consequences of having made a real monetary donation. We expect that (compared to younger adults), older adults will draw more positive affect from their donations and feel less negative affect.

### Method

#### Participants

One-hundred and eight participants from Gothenburg University participant pool, consisting of people from the community that signed up on a voluntary participant list, were sent a letter asking them about a donation decision that they 5 days earlier made in a previous study. A final sample of 72 participants, age range 19–89 years, *M*_age_ = 49 years, 68% females, returned the survey. Of participants 2% were teenagers, 33% were in their 20s, 6% were in their 30s, 2% were in their 40s, 21% were in their 50s, 18% were in their 60s, 15% were in their 70s, and 4% were in their 80s.

#### Design and Procedure

After completing an unrelated study at the university, participants were told that they could get 90 SEK (Swedish Kroner; equivalent to $15) as compensation for their time. They were also told that if they preferred, they could give away a part or all of this money to a charity. We presented them with a picture of a child in need and informed participants that “This child is in need of aid. The child is facing starvation and is in immediate need of food.” Their donation decision constituted the first part of this experiment.

Five days later, the participants were sent a letter asking them about their donation and how they felt about their choice of donating or not. We again presented them with the picture of the child and stated the sum donated to the child. We thanked them for participating in the experiment and asked them to think about their donation and their emotions. Participants were instructed to put the survey in a pre-paid letter and sent it back to us.

#### Measures

We had information about their age and how much of their compensation they actually gave to charity (0–90 SEK) in the donation decision. In addition, we also asked them how happy, sad, and how much “warm glow” they felt when thinking about their choice on a five-point scale (1 = not at all, 5 = very much).

### Results

First, we wanted to investigate whether the participants in the study had donated any money to charity. Our analysis concluded that 40% of the participants donated at least some of their reward to charity, and of those 17% donated all of their reward, the mean amount donated was 22.2 SEK ($3.4) with a standard deviation of 33.9 SEK ($5.3).

To investigate if there were any differences between older and younger adults regarding how much money they donated, we looked at the correlation between the amount donated and the participants’ age, and due to the non-normal distribution of donations, we used Spearman’s rank order correlation coefficient (i.e., Spearman’s rho). The analysis showed that the age of the participant did not correlate with the amount donated to charity [*rs*(72) = 0.209, *p* = 0.08], however, a slight trend was seen indicating that older adults might donate more.

To investigate the feelings elicited when donating money to charity, we asked participants to think about their choice of donating, and to rate the feelings (happy, sad and “warm glow”) elicited while doing so on a five-point scale. The analysis showed, in line with the positivity bias, that older participants, whether they donated money or not, felt more “warm glow” [*r*(70) = 0.263, *p* = 0.01] when thinking about their choice, however, age had no impact on sadness [*r*(70) = -0.14, *p* = 0.24] and only a marginal effect on happiness [*r*(70) = 0.21, *p* = 0.08]. These effects were seen independently if the participants donated or not.

More importantly to our research question, wanted to investigate if older adults differed from younger adults in the emotions elicited from donating money to charity. We expected that older adults that donated money would benefit more from this donation than younger adults, due to the positivity bias. To investigate this, we divided the participants into two groups: participants that donated nothing or less than half of their reward and participants that donated more than half of their reward. In a regression analysis we investigated whether or not age and donating money interacted on happiness, sadness and “warm glow.” Our first regression analysis [*R*^2^ = 0.12, *F*(3,68) = 3.19, *p* = 0.03] revealed a significant interaction suggesting that older adults who donated felt happier (*b* = 0.75, *SE* = 0.34, *p* = 0.01) than younger that donated [simple slopes for participant that did not donate *b* = 0.000, *SE* = 0.004, *t*(68) = 0.08*, p* = 0.93, for participants that did donate *b* = 0.020, *SE* = 0.001*, t*(68) = -2.16, *p* = 0.93]. This demonstrated that older adults have a stronger positive reaction to donating money to charity compared to younger adults. Our second regression analysis [*R*^2^ = 0.14, *F*(3,68) = 3.59, *p* = 0.02] revealed a non-significant but marginal interaction suggesting that older adults who donated might feel more “warm glow” [*b* = 0.61, *SE* = 0.37, *p* = 0.10] compared to younger adults that donated [simple slopes for participant that did not donate *b* = 0.001*, SE* = 0.007, *t*(68) = 0.08*, p* = 0.21, for participants that did donate *b* = 0.04, *SE* = 0.016, *t*(68) = 2.41, *p* = 0.02]. Our final regression analysis revealed a significant interaction suggesting that older adults who donated felt less sadness (*b* = -0.43, *SE* = 0.21, *p* = 0.04). the simple slope analyses showed similarly to the other analyses that participant that did not donate had a non-significant simple slope (*b* = 0.000, *SE* = 0.004, *t*(68) = 0.08*, p* = 0.93), however, participants that did donate had a significant simple slope [*b* = -0.021, *SE* = 0.010*, t*(68) = -2.17, *p* = 0.03]. However, the general model [*R*^2^ = 0.07, *F*(3,68) = 1.58, *p* = 0.20] was not significant and the *R*^2^ low, which indicates a poor model fit and this last estimate should be interpreted with caution.

### Discussion

In this study, we showed that there were differences between younger and older adults in the emotional consequences of making a monetary donation. As anticipated from the positivity bias, older adults report more positive affect overall compared to younger adults. Hence, older adults feel more positive affect when thinking about their prosocial decisions, regardless of whether or not they actually donated any money. This may be consistent with the finding that older adults are especially motivated to process the positive above the negative details of their personal experiences in order to attain or maintain a positive state of mind (Mather and Carstensen, 2005). An alternative explanation is that older adults, to a greater extent than younger adults, avoid remembering negative affect, as they try to avoid thinking about things that elicit negative affect.

Importantly, donating further increased the emotional consequences for both younger and older adults. However, older adults, who donated, felt more happiness and “warm glow” when thinking about their donation compared to younger adults (who also donated money). It seems that the positive affective consequences of donating money are stronger in the older adults of our sample and therefore older adults may gain more from donating money to charities than do younger adults.

Finally, unlike Study 1, older adults donated significantly more money to the person in need. Part of the difference could be that this donation constituted a real monetary donation from money that they got from a previous study. Moreover, our results give additional support to the idea that generosity increases with age (Midlarsky and Hannah, 1989; McAdams et al., 1993). We also conclude that the participants who gave away their money to a person in need were the people gaining the most positive affect from the compensation when asked about the money a few days after they received it.

## General Discussion

The present studies show that older adults (compared to younger adults) derive more positive affect from acting prosocially, both when it comes to the decision process before the donation but also when it comes to the emotional outcome the decision. Moreover, these findings are consistent with research showing lower levels of negative affect, and higher levels of positive affect, in older adults (Carstensen et al., 2000). The present research suggests that the emotional differences between older and younger adults have influences on how everyday behaviors such as acting prosaically are made and have an influence on the outcomes of those behaviors. An important finding in the present research was that prosocial behavior in older adults is less motivated by negative emotions when compared to younger adults. This indicates that even though negative emotions can be a consequence of seeing someone in need, there are age related differences in the judgments and decisions connected to these emotions. Carstensen and Mikels (2005) suggests that these age related differences in emotion originates from strategies that older adults use to maximize the positive outcome from a given event.

Previous research demonstrating a relationship between monetary donations and overall happiness has shown that ‘doing good’ is associated with ‘feeling good’ (Dunn et al., 2008). It is therefore likely that the differences in emotional responses to prosocial behavior similar to what has been shown in the present research will have implications for overall well-being. However, not only does it seem like helping others has a beneficial influence on one’s feelings, doing good, and frequent volunteering in particular, may have more direct effects on health (Kim and Ferraro, 2013). Our results show that older adults gain more from prosocial behavior than younger adults. However, prosocial behavior can have both negative as well as positive aspects. Resent research have found that older adults are motivated not only by positive aspects but especially by low arousal positive aspects (i.e., satisfaction rather than excitement), it is therefore important to investigate what forms of prosocial behavior that fits older adults motivational needs (Bjälkebring et al., 2015; Sands and Isaacowitz, 2016).

Some limitations of the present research should be acknowledged: (1) The approach of this paper was to investigate processes behind prosocial giving, hence the studies was set up in a way to facilitate giving from the participants. The questions about feelings such as warm glow as well as other aspects could have increased overall donations. Hence, we don’t know if a similar pattern would emerge in a naturalistic setting. Study 1 used fictional donations and may therefore have resulted in a higher level of donations compared to real donations. However, hypothetical donations have been shown to be a good proxy for real donations (e.g., Kogut and Ritov, 2005). (2) More generally, the type of cross-sectional comparisons used here cannot rule out cohort effects. Therefore these results should only be interpreted as differences between younger and older adults, not as aging effects *per se*. Bekkers and Wiepking (2011) also state that there are more reasons to donate that just emotional reasons, the age related emotional differences can explain some age related differences in donation behavior, however, there are most likely other age related differences that are related to donations, such as income. (3) Another potential limitation is that the constructs in this paper are assessed with single items. While single items can be successfully used to measure constructs that are sufficiently narrow, future studies should also assess perceived impact, mental imagery, and feelings with multiple-item scales to increase reliability. (4) Finally, we acknowledge that the effects of age on donation probably do not supersede other relevant factors that influence charitable giving. Naturally, for example, age related differences in motivation and emotional processing might be less important when financial constraints are present (Dickert et al., 2011a).

## Conclusion

To conclude, helping others is typically associated with both negative and positive emotions. However, the present research suggest that age-related changes in affective processing related to real judgment and decisions may protect from the negative consequences, and increases the positive consequences, of helping others. Older adults both show benefit from giving in both the decision process and in the emotional outcome of the decision to donate. In addition, older adults show less motivation from negative affect, which suggest that they avoid process negative emotions and are motivated rather by positive emotions related to donations. Consequently, to increase the well-being in the second half of the lifespan, older and younger adults should be encouraged to help others and given opportunities to do so.

## Author Contributions

PB, DV, SD, and PS developed the study concept and the study design. Testing and data collection were performed by PB as well as DV. PB performed the data analysis and interpretation with help by DV and SD. PB and SD drafted the manuscript, and DV and PS provided critical revisions. All authors approved the final version of the manuscript for submission.

## Conflict of Interest Statement

The authors declare that the research was conducted in the absence of any commercial or financial relationships that could be construed as a potential conflict of interest.
