# Supplementary material for: Greater Emotional Gain from Giving in Older Adults: Age-Related Positivity Bias in Charitable Giving
Source: Front Psychol. 2016 Jun 15;7:846. doi: 10.3389/fpsyg.2016.00846 (PMC4908114; doi:10.3389/fpsyg.2016.00846)
Supplement: Supplementary file 1 [file Table_1.PDF]

Supplementary Material

Greater emotional gain from giving in older adults: Age-related positivity bias in charitable giving

Pär Bjälkebring 1*, Daniel Västfjäll 2,4, Stephan Dickert 2 3, Paul Slovic 4.

[1] University of Gothenburg, Department of Psychology, Gothenburg, Sweden

[2] Linköping University, Linköping, Sweden

[3] Vienna University of Economics and Business, Vienna, Austria

[4] Decision Research, University of Oregon, Eugene, Oregon, USA

*** Correspondence:** Pär Bjälkebring, Department of Psychology, Haraldsgatan 1, Gothenburg, 41314 , Sweden

par.bjalkebring@psy.gu.se

Supplementary Figures and Tables

A factor analysis was performed to see how the positive as well as negative items factored together. As all items were directed to the same target, the child in need, they had higher correlations compared to what would be expected in other situations.

To allow for a separation into components a visual inspection of a scree plot was used. After three factors the scree plot flattened out. The third component explained about 9% of the variance (see Table 1) and together the three components explain 95% of the variance.

As seen in Table 3, Sad, Sympathy and Upset formed one factor, compassion and sympathy formed one factor and warm glow formed one factor on its own.

Rotation was performed with a varimax rotation.

Table 4 show that removing warm glow from the composite of positive emotions comes to the same insignificant results.

Component	Extraction Sums of Squared Loadings		
	Eigenvalue	% of Variance	Cumulative %
1	4,543	75,721	75,721
2	,613	10,225	85,946
3	,527	8,789	94,735

Table 1. Extraction Sums of Squared Loadings

	Component		
	1	2	3
I felt sad	,932	-,116	,012
I felt sympathy	,903	-,015	-,390
I felt compassion	,902	,021	-,380
I felt upset	,900	-,218	,295
I felt worried	,884	-,205	,329
I felt warm glow	,674	,714	,189

Table 2. Component matrix, Extraction Method: Principal Component Analysis. 3 components extracted.

	Component		
	1	2	3
After seeing M'bago, I felt worried (child alone)	**,879**	,329	,226
After seeing M'bago, I felt upset (child alone)	**,875**	,365	,211
After seeing M'bago, I felt sad (child alone)	**,684**	,597	,243
I felt sympathy toward M'bago(child alone)	,380	**,880**	,220
I felt compassion toward M'bago (child alone)	,370	**,870**	,255
Warm glow for child alone condition	,247	,255	**,935**

Table 3. Rotated Component Matrix, Rotation Method: Varimax with Kaiser Normalization. Rotation converged in 5 iterations.

Table 4.

	Positive Emotions With Warm Glow				Positive Emotions Without Warm Glow			
	β	*b*	*SE*	*p*	β	*B*	*SE*	*p*
Age	-.07	-.091	.059	.12	-.09	-.12	.063	.051
Emotion	.056	10.322	.821	<.001	.048	7.85	.776	<.001
Interaction	-.02	-.026	.076	.73	-.02	-.035	.068	.61
R2		.31	-	<.001		.23	-	<.001
